# Chloral Hydrate as a Vesicant

**Published:** 1884-08

**Authors:** 


### Editorial Notes.

It is said to have been proved that the cholera was imported into France by the French troops ships which had just arrived from the East (Tonquin Cochin China).

Chloral Hydrate as a Vesicant.—It is stated that powdered chloral hydrate, sprinkled upon adhesive plaster, which is then sufficiently heated to cause it to adhere to the skin, and immediately applied to the surface where a blister is desired, will as effectually accomplish the purpose in ten minutes as a cantharidal plaster will in six hours. The pain produced by it is but slight.

An ingenious application of the condom in the treatment of epistaxis was suggested by the late Prof. McDowell. For the purpose of arresting the hemorrhage the condom is tied to the end of a small flexible catheter and a piece of rubber tubing, with a clamp, connected with the catheter. The condom is then lubricated and introduced into the nostril from which the bleeding proceeds, and when in place inflated with air or water as desired.

A correspondent of the *Times* says that the treatment of cholera in Marseilles and Toulon is as follows: In the first stage twenty drops of laudanum are given with three grammes of ether, and ice in the mouth to stop the vomiting. In the second stage, from ten to fifteen grammes of acetate of ammonia, the same quantity of alcohol and injections of morphia are given. If the patient has embarrassed breathing, oxygen is inhaled and the limbs are rubbed with turpentine. The third stage is the coffin. At first nineteen-twentieths of the patients died—later the mortality became less frightful.

